# A systematic review of health state utility values and psychometric performance of generic preference-based instruments for children and adolescents with mental health problems

**DOI:** 10.1007/s11136-023-03441-x

**Published:** 2023-05-26

**Authors:** Thao T. H. Thai, Lidia Engel, Joahna Kevin Perez, Eng Joo Tan, Sandra Eades, Lena Sanci, Cathrine Mihalopoulos

**Affiliations:** 1 Monash University Health Economics Group, School of Public Health and Preventive Medicine, Monash University, Level 4, 553 St Kilda Road, Melbourne, VIC 3004 Australia; 2 Centre for Epidemiology and Biostatistics, School of Population and Global Health, Faculty of Medicine, Dentistry and Health Sciences, The University of Melbourne, 780 Elizabeth Street, Melbourne, VIC 3000 Australia; 3 Department of General Practice, Melbourne Medical School, Faculty of Medicine, Dentistry and Health Sciences, The University of Melbourne, 780 Elizabeth Street, Melbourne, VIC 3000 Australia

**Keywords:** Health state utility values, Children and adolescents, Mental health problems

## Abstract

**Aims:**

This paper aims to systematically identify reported health state utility values (HSUVs) in children and adolescents with mental health problems (MHPs) aged less than 25 years; to summarise the techniques used to elicit HSUVs; and to examine the psychometric performance of the identified multi-attribute utility instruments (MAUIs) used in this space.

**Methods:**

A systematic review was conducted following PRISMA guidelines. Peer-reviewed studies published in English, reporting HSUVs for children and adolescents with MHPs using direct or indirect valuation methods were searched in six databases.

**Results:**

We found 38 studies reporting HSUVs for 12 types of MHPs across 12 countries between 2005 and October 2021. Attention deficit hyperactivity disorder (ADHD) and depression are the most explored MHPs. Disruptive Behaviour Disorder was associated with the lowest reported HSUVs of 0.06 while cannabis use disorder was associated with the highest HSUVs of 0.88. Indirect valuation method through the use of MAUIs (95% of included studies) was the most frequently used approach, while direct valuation methods (Standard Gamble, Time Trade-Off) were only used to derive HSUVs in ADHD. This review found limited evidence of the psychometric performance of MAUIs used in children and adolescents with MHPs.

**Conclusion:**

This review provides an overview of HSUVs of various MHPs, the current practice to generate HSUVs, and the psychometric performance of MAUIs used in children and adolescents with MHPs. It highlights the need for more rigorous and extensive psychometric assessments to produce evidence on the suitability of MAUIs used in this area.

**Supplementary Information:**

The online version contains supplementary material available at 10.1007/s11136-023-03441-x.

## Introduction

Mental health problems (MHPs) denote a worldwide public health challenge and one of the leading causes of disability in children and youth population [1, 2]. With the prevalence being around 14% worldwide, MHPs often account for a high global burden of disease (13% of disability-adjusted life-years) [3]. MHPs often persist to adulthood, impacting not only the health and well-being of children and youth but also their socioeconomic trajectories and family life [4]. Depression, anxiety and behavioural disorders are among the leading causes of disability while suicide is one of the leading causes of death among youths [3].

Economic evaluation is increasingly used to inform decision-making in priority setting worldwide, and is often recommended by government agencies [5]. Economic evaluations using cost-utility analyses (CUAs) commonly require the estimation of quality-adjusted life-years (QALYs) to capture the benefits resulting from health interventions. QALYs are calculated by combining length of life and health-related quality of life (HRQoL), where the time spent in a particular health state is weighted by corresponding health state utility values (HSUVs)—used to denote the “quality” in QALYs [6]. HSUVs represent the strength of preference for a particular health state and are anchored on a cardinal scale between 0 (dead) and 1 (full health), where full health is usually defined by the domains of HRQoL measured on each instrument [7].

HSUVs can be derived using either direct or indirect valuation methods [6]. Direct methods include choice-based valuation methods, such as standard gamble (SG), time trade-off (TTO), discrete choice experiments, or best–worst scaling to value health state description (vignettes) or individual’s health state. Indirect valuation method involves the use of generic multi-attribute utility instruments (MAUIs) which usually comprise a descriptive system (largely health related quality of life questionnaires) describing various dimensions of HRQoL, and a preference-based scoring algorithm (tariff) used to convert responses from the descriptive system to a single, numeric HSUV [7]. MAUIs are available as self-report (i.e. one assesses one’s own health state) or proxy-report (i.e. one assesses the health state experienced by someone else) [7].

In modelling-based economic evaluations, analysts may not have the resources and time to acquire original HSUVs for health states of interest, thus often relying on systematic reviews on HSUVs as a robust and transparent source of evidence for various health conditions. Furthermore, HSUVs can be used to explore the burden of disease, especially when compared to population norms or people without the disorder in question. To the best of our knowledge, there has been no previous systematic review on HSUVs for children and youth with MHPs. Furthermore, with the reorientation of mental health services aimed at the young population, which is widely defined as those aged between 15 and 25 years [8], an age group that often experiences a high rate of MHPs [9], exploring HSUVs for the age range up to 25 years is helpful to support studies aimed at this age group which would otherwise be unintentionally excluded by the artificial cut-off point of being 18 years old between adolescents and adults [10].

Over the last decade, while there has been an increased focus on the valuation methods using MAUIs to produce HSUVs for children and youth, the guidelines from international agencies for assessing health outcomes in this population remain unclear [10, 11]. This is concerning as an adequate measure to capture healthcare interventions’ benefits plays a crucial role in the robustness, transparency and rigour of economic evaluations. In adult population, it is well reported that some commonly used MAUIs lack sensitivity to measure the impact of MHPs on quality of life [12]. Specifically, there is evidence that the most commonly used MAUIs, such as the EQ-5D and SF-36 are not sufficiently sensitive to reflect the impact of severe MHPs such as schizophrenia [13, 14], or bipolar disorder [15]. Meanwhile, the psychometric performance of MAUIs used in children and youth with MHPs is not well explored.

The aims of this paper are three-fold: (i) to identify reported utility values associated with MPHs in children and youth aged less than 25 years and conduct a meta-analysis of reported HSUVs if appropriate; (ii) to summarise the elicitation techniques used to derive utility values among children and youth with MPHs; and (iii) to provide a summary of the evidence on the psychometric performance of the identified MAUIs used in this space.

## Methods

### Search strategy

A systematic literature review was conducted following the Preferred Reporting Items for Systematic Review and Meta-Analyses (PRISMA) checklist [16]. Systematic searches without time limit were conducted in MEDLINE (via Ovid), CINAHL (via EBSCOhost), PsycINFO (via EBSCOhost), Embase (1947 onwards), EconLit (1886 onwards), Cochrane Library (including the Health Technology Assessment Database and NHS Economic Evaluation Database) in October 2021. Search terms (Supplementary Document 1) were developed based on main concepts of (a) HSUVs, (b) direct preference elicitation methods, (c) indirect preference elicitation methods, (d) population of interest, and (e) MHPs. A search strategy that included both keywords and Medical Subject Headings (MESH) terms was developed for MEDLINE (Ovid) and subsequently translated to other databases (Supplementary Document 1). Covidence program was used for duplicate removal and for the screening process. Titles and abstracts were completed by three independent reviewers (TT, JP and AT). If an article received two positive votes, it moved to the next stage of full-text screening. In case of conflicts, a third reviewer was referred to make the final assessment. The same process occurred in the full-text screening.

### Inclusion and exclusion criteria

(i) *Type of study*: (a) observational and experimental design studies estimating HSUVs in children and youths with MHPs using either direct or indirect elicitation methods; (b) trial-based or model-based economic evaluations, describing the use of HSUVs in CUA of interventions addressing MHPs for children and youths.
(ii) *Study population*: Child and young populations (aged up to 25 years) with any MHPs with diagnostic assessment or screened by a self-reported measure such as Kessler-10. Autism-related studies were excluded following national classifications, which do not classify autism as an MHP [17, 18]. As the review focused on studies reporting HSUVs for MHPs, we excluded studies reporting HSUVs for comorbidities with MHPs (e.g., heart disease and depression).
(iii) *Type of respondents*: proxy- or self-reported HSUVs were included.
(iv) *Type of publication:* peer-reviewed studies and written in English. Grey literature (e.g., conference abstracts and proceedings, discussion papers, reports, and unpublished theses) were excluded. Articles that reported HSUVs derived from non-generic MAUI (if using indirect preference elicitation methods) were excluded. Previous relevant systematic reviews [19, 20] were used for manual article searching.

### Data extraction

Data extraction was conducted by TT and checked by two other authors (AT and JP). The following data were extracted:

1. *Study description*: lead author, publication year, country of publication, sample size, study type.
2. *Study population characteristics:* age range, mental health condition. We classified the age groups following the recommended practice from the Australian government as: early childhood (< 5 years), primary school children (5–12 years), adolescence (12–19 years) and young adults (20–25 years) [21].
3. *Direct valuation method*: method used, proxy- or self-report, reported mean and standard deviation of HSUVs.
4. *Indirect valuation method*: generic MAUIs used, proxy- or self-report, algorithm applied, reported mean and standard deviation HSUVs.

For studies reporting HSUVs at different time points, we only extracted baselines scores. For studies reporting HSUVs for more than one type of MHPs, HSUVs for each MHPs were extracted with the corresponding sub-samples. Meta-analysis of reported HSUVs was only conducted if reported HSUVs for the same health condition were derived from the same population using the same valuation method with the same country-specific algorithms [22].

### Assessment of psychometric performance

Assessment of the psychometric performance of MAUIs was based on its reliability, validity, and responsiveness as outlined in Rowen et al. [23]. Data were extracted about the following information if available.

1. *Name* of MAUIs used, algorithms used, whether the MAUI was self-reported and/or proxy-reported by parents/caregivers, health professionals.
2. *Study sample* type of mental health problems studied, sample size, and age range.
3. *Known-group or discriminant validity* refers to the ability of an instrument to reflect known-group differences (e.g. with and without the condition).
4. *Convergent validity* refers to the extent to which an instrument converges with other measures of the same concept (i.e., the strength of association with other measures of the same concept).
5. *Responsiveness* is the capacity of a MAUI to reliably detect changes in HRQoL because of a change in their health status.
6. *Reliability* reflects the ability of an instrument in reproducing unchanged utility values where no change in health is detected when measured (a) at two different time points (test–retest reliability), (b) using different administration modes (intermodal reliability), (c) by different respondents (interrater reliability) [23].
7. *Acceptability and feasibility* are assessed by the proportion of missing answers and respondents’ understanding of the measure.
8. *Internal consistency reliability* explores whether items within a MAUI are measuring the same construct.

## Results

### Search results

**Fig. 1:**
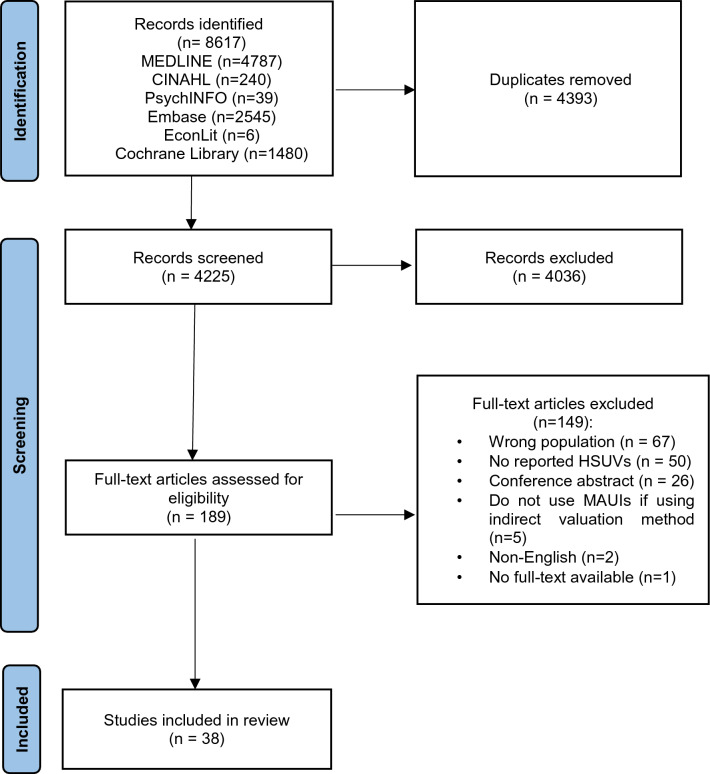
PRISMA diagram

After deduplication, 4225 articles were included for the title and abstract screening. Based on the selection criteria, we screened 189 full texts and of these, 38 were included for data extraction. A PRISMA flow chart of the study selection process is provided in Fig. 1.

### Study characteristics

Table 1 summarises the characteristics of 38 included articles that cover 12 types of MHPs from 2005 to 2021 (Supplementary document 2 for more information). Most studies (89%) were conducted between 2010 and 2021, which indicates a growing research interest in the burden associated with youth mental health. Of 38 included studies, 20 were cross-sectional studies and 18 were randomised controlled trials.

**Table 1:** Study characteristics and reported utility values by type of mental health conditions

First author, year (Reference)	Country	Type of conditions	Type of study	N	Age range (mean age)	Instruments	Algorithm	Perspectives	Utility scores Mean (SD)
1. ADHD									
Matza, 2005 [27]	US	ADHD	Cross-sectional	43	5–17 (10.2)	SG	NA	Proxy-report (parents)	Mild ADHD 0.71 (SD 0.25); Moderate ADHD 0.58 (SD 0.27); Severe ADHD 0.48 (SD 0.28)
Secnik, 2005 [30]	UK	ADHD	Cross-sectional	83	7–18 (12.6)	SG, EQ-5D-3L	EQ-5D-3L: UK	Proxy-report (parent)	Child’s own current health state: SG 0.72 (0.21) EQ-5D-3L: 0.74 (0.19)
Lloyd, 2011 [66]	UK	ADHD	Cross-sectional	100	8–16 (11)	TTO	NA	Proxy-report (general public)	Borderline to mildly ill: 0.787 (0.217) Moderately to markedly ill 0.578 (0.275) Severely ill 0.444 (0.230)
Matza, 2005 [61]	US and UK	ADHD	Cross-sectional	43 (US) and 83 (UK)	7–18 (11.8)	EQ-5D-3L	UK	Proxy-report (parent)	Total sample: 0.75 (0.17) US sample: 0.78 (0.15) UK sample: 0.74 (0.19)
Peasgood, 2016 [29]	UK	ADHD	Cross-sectional	476	6–18 (11.8)	EQ-5D-Y, CHU9D	CHU9D: UK (adults)	Self-report	EQ-VAS 80.16 (SD 20.61); CHU9D 0.82 (SD 0.12) EQ-5D-Y: not reported
Bouwmans, 2014 [40]	Netherlands	ADHD	Cross-sectional	739	6–18 (11.9)	EQ-5D-3L	Dutch (adults)	Proxy-report (parent)	0.81 (0.17)
Maia, 2016 [58]	Brazil	ADHD	A naturalistic study	62 children and 28 adolescents	6–17 Children mean age = 8.62, Adolescent mean age = 13.67)	HUI2 and HUI3	Canada tariff	Proxy-report (parent)	Children: 0.69; Adolescents: 0.66
Sayal, 2016 [31]	UK	At risk of ADHD	RCT	55 teachers and 40 parents	4–8(6.16)	EQ-5D-Y and CHU9D	EQ-5D-Y: UK (adults) CHU9D: UK (adults)	Proxy-report (parent and teacher)	Control arm: EQ-5D-Y 0.815 (0.257), CHU9D 0.847 (0.099); Parent only arm: ED-5D-Y 0.734 (0.370) CHU9D (0.818 (0.128); Parent + Teacher arm: EQ-5D-Y 0.771 (0.294); CHU9D 0.897 (0.102)
Le 2021 [24]	Australia	ADHD	Cross-sectional	160	11–17	CHU-9D	Australia (adolescents)	Self-report	0.74 (0.19)
Petrou, 2010 [25]	UK and Republic of Ireland	ADHD	Cross-sectional	331 term-born classmates	5–11	HUI2&HUI3	HUI3: Canada (adults) HUI2: UK (adults)	Proxy report (parents)	HUI3: 0.629 (0.271); HUI2: 0.792 0.120
2. Anxiety disorder									
Bodden, 2008 [41]	Netherlands	Anxiety disorder (except for obsessive–compulsive disorder and post-traumatic stress disorder	RCT	116	8–17 years (12.3)	EQ-5D-3L	UK (adults)	Self-report	Control: 0.87 (0.13), Intervention: 0.83 (0.20)
Creswell, 2017 [32]	UK	Anxiety disorders	RCT	136	5–12 years	CHU9D	UK tariff	Self- and proxy-report (parent)	Self-reported: CHU9D: Control 0·87 (0·09); Intervention 0·88 (0·09) EQ-5D-Y: Control 0·82 (0·15); Intervention 0·80 (0·20) Proxy-reported: CHU9D: Control 0·85 (0·10); Intervention 0·89 (0·07)
Chatterton, 2019 [53]	Australia	Anxiety disorder	RCT	281	7–17	CHU9D	Australia (adolescents)	Self-report	Control: 0.62 (0.24), Intervention 0.64 (0.21)
Le 2021 [24]	Australia	Anxiety disorder	Cross-sectional	97	11–17	CHU-9D	Australia (adolescents)	Self-report	0.70 (0.24)
Astrom, 2020 [26]	Sweden	Anxiety disorder	Cross-sectional	13	13–17 (15.4)	EQ-5D-Y-5L and EQ-VAS	NA	Self-report	EQ-VAS: 23.5 (15.8) EQ-5D-Y-5L: not reported
3. Depression									
Lynch, 2016 [56]	US	Depression	Cross-sectional	169	13–17 (15.4)	HUI2 and HUI3, EQ-5D-3L, QWB, SF6D	HUI2&3: Canada EQ-5D-3L QWB SF-6D: UK general population	Self-report	HUI-2: 0.695 (SD 0.183); HUI3 0.545 (SD 0.266); EQ-5D 0.759 (SD 0.154); QWB 0.574 (SD 0.090); SF-6D 0.645 (SD 0.087)
Turner, 2017 [33]	UK	Depression	RCT	86	14–17	EQ-5D-5L	UK	Self-report	Control 0.82 Intervention 0.74
Dickerson, 2018 [57]	US	Depression	Cross-sectional	376	13–17	(EQ-5D-3L), (HUI2) and (HUI3), (QWB), (SF-6D)	HUI2&3: Canada EQ-5D-3L QWB SF-6D: UK general population	Self-report	HUI2 0.73 (0.18); HUI3 0.56 (0.27); EQ-5D-3L 0.81 (0.15); QWB 0.60 (0.09); SF-6D 0.67 (0.09)
Byford, 2013[35]	UK	Depression	RCT	199	11–17	EQ-5D-3L	UK adult tariff	Self-report	0.495 (SD 0.296)
Byford, 2007 [34]	UK	Depression	RCT	208	11–17	EQ-5D-3L	UK adult tariff	Self-report	Intervention: 0.49 (0.30); Control: 0.50 (0.29)
Wright, 2017 [36]	UK	Depression	RCT	91	12–18	EQ-5D-Y, HUI-2		Self-report	EQ-5D-Y: Intervention: 0.53 (0.26); Control 0.60 (0.33); HUI-2: not reported
Goodyer, 2017 [37]	UK	Depression	RCT	447	11–17	EQ-5D-3L	UK adult tariff	Self-report	Intervention 1: 0.596 (0.275); Intervention 2: 0.578 (0.281); Intervention 3: 0.569 (0.258)
Le 2021 [24]	Australia	Major depressive disorder	Cross-sectional	159	11–17	CHU9D	Australia (adolescents)	Self-report	0.64 (0.26)
Astrom, 2020 [26]	Sweden	Depressive episode/recurrent depressive disorder	Cross-sectional	15	13–17 (15.4)	EQ-5D-Y-5L and EQ-VAS	NA	Self-report	EQ-VAS: 28.2 (14.4) EQ-5D-Y-5L: not reported
4. Undefined psychiatric disorders									
Petrou, 2010 [25]	UK and Republic of Ireland	Psychiatric disorders	Cross-sectional	50	5–12	HUI2&HUI3	HUI3: Canada (adults) HUI2: UK (adults)	Proxy report (parents)	HUI3: Any DSM–IV clinical diagnosis 0.698 (0.273); HUI2: Any DSM–IV clinical diagnosis 0.782 (0.149)
Ougrin, 2018 [39]	UK	Psychiatric inpatients	RCT	106	12–17	EQ-5D-3L	UK (adults)	Self-report	Intervention: 0.5 (0.3) Control: 0.6 (0.4)
Astrom, 2020 [26]	Sweden	Psychiatric inpatients	Cross-sectional	52	13–17 (15.4)	EQ-5D-Y-5L and EQ-VAS	NA	Self-report	All disorders by age group 13–17 years: 29.2 (19.5) 13–15 years: 25.3 (19.4) 16–17 years: 32.6 (19.3) Bipolar affective disorder: 28.3 (27.7) Reaction to severe stress/ adjustment disorders: 42.5 (23.2) Other: 23.1 (15.1) Observation for suspected mental and behavioural disorders: 39.0 (28.6)
5. Internalizing problems									
Philipsson, 2013 [50]	Sweden	Internalizing problems with recurrent visits to the school nurse for psychosomatic symptoms	RCT	112 girls	13–18	HUI 3		Self-report	Intervention: 0.71 (0.66– 0.77) Control: 0.77 (0.73–0.82)
6. Personality disorders									
Swales 2016 [38]	UK	Borderline personality disorder	Cross-sectional	76	14–18	EQ-5D-3L	UK tariff	Self-report (some used clinicians as proxy)	0.236 (SD 0.32)
Feenstra, 2012 [63]	Netherlands	Personality disorders	Cross-sectional	131	14–19 (16.6)	EQ-5D-3L	Dutch tariff	Self-report	Total: 0.55 (SD 0.27); Borderline personality disorder 0.49 (0.28), Avoidant personality disorder 0.49 (0.27), Personality disorder not otherwise specified 0.70(0.23), Depressive personality disorder 0.34(0.20), Obsessive–compulsive personality disorder 0.50 (0.33)
7. Behaviour disorders									
Vermeulen, 2017 [43]	Netherlands	Oppositional Defiant Disorder (ODD), Conduct Disorder (CD), Disruptive Behaviour Disorder (DBD)	Cross-sectional	Professionals, children, adolescents and parents	4–18	EQ-5D-3L	UK tariff	Proxy report (parents, professionals and children)	Professionals: Children: ODD 0.58 (0.08), CD 0.47 (0.14), DBD 0.58 (0.08), Adolescents: ODD 0.59 (0.12), CD 0.58 (0.15), DBD 0.59 (0.12); Parents: Regular primary education (children’s parents): ODD 0.52 (0.080), CD 0.36 (0.125), DBD 0.28 (0.122) Regular secondary education (adolescents’ parents): ODD 0.49 (0.12), CD 0.23 (0.12), DBD 0.12 (0.12), Vocational training (parents of adolescents with behaviour disorders) ODD 0.485 (0.14), CD 0.269 (0.13), DBD 0.190 (0.14); Children and adolescents: Regular primary education (children) ODD 0.31 (0.15), CD 0.11 (0.09), DBD 0.06 (0.07), Regular secondary education (adolescents) ODD 0.52 (0.12), CD 0.31 (0.13), DBD 0.20 (0.13), Vocational training (adolescents) ODD 0.37 (0.14), CD 0.19 (0.15), DBD 0.12 (0.15) Adolescents in vocational training (with behavioural disorders): own valuation 0.84 (0.008)
Le 2021 [24]	Australia	Conduct disorder	Cross-sectional	48	11–17	CHU-9D	Australia (adolescents)	Self-report	0.71 (0.23)
Petrou, 2010 [25]	UK and Republic of Ireland	Emotional disorder; Conduct disorder	Cross-sectional	Emotional disorder n = 16 Conduct disorder n = 17	5–12	HUI2&HUI3	HUI3: Canada (adults) HUI2: UK (adults)	Proxy report (parents)	HUI3: Any emotional disorder 0.672 (0.296); Any conduct disorder 0.727 (0.260); HUI2: Any emotional disorder 0.760 0.161; Any conduct disorder 0.802 0.129;
8. Post-traumatic stress disorder									
Dams, 2021 [64]	Germany	PTSD	RCT	87	14–21 (18.1)	EQ-5D-5L	German (adults)	Self-report	0.70 (0.25)
Dams, 2020 [52]	Germany	PTSD	RCT	87	14–21	EQ-5D-5L	German (adults)	Self-report	0.70 (0.03)
Aas, 2019 [60]	Norway	PTSD	RCT	156	10–18	16D	Finland	Self-report	Intervention: 0.755 (0.012); Control: 0.777 (0.104)
9. Substance use disorders									
Goorden, 2015 [44]	Netherlands	Cannabis use disorder	RCT	96 (Control 49; Intervention 47)	13–18	EQ-5D-3L	Dutch (adults)	Self-report	Intervention: 0.88 (0.15) Control: 0.89 (0.13)
Reckers-Droog, 2020 [45]	Netherlands	Adolescents receiving in- or outpatient mental health care with substance use	Cross-sectional	167	12–18 (15.2)	EQ-5D-3L	Dutch (adults)	Self-report	Alcohol use 0.82 (0.23); Drugs use 0.83 (0.18); Medicine use 0.81 (0.18); Delinquency 0.82 (0.23)
10. Psychosis									
Grano, 2014 [48]	Finland	At risk for psychosis	Cross-sectional	202	11–22 (15.6)	16D	Finland	Self-report	Not at heightened risk of developing psychosis: 0.87 2(0.091) At heightened risk of developing psychosis: 0.795 (0.106)
Grano, 2013 [47]	Finland	At risk for psychosis	Cross-sectional	Total: 90 (Not at heightened risk n = 55; At heightened risk n = 35)	12–21 (15.1)	16D	Finland	Self-report	Not at heightened risk of developing psychosis: 0.8737 (0.0931) At heightened risk of developing psychosis: 0.7992 (0.1)
Grano, 2015 [46]	Finland	At risk for psychosis	Cross-sectional	181 (At risk n = 57 Help-seekers n = 124)	12–22 (15.3)	16D	Finland	Self-report	At risk: 0.80 (0.10) Help-seekers (not at risk): 0.87 (0.09)
11. Self-harm									
Tubeuf, 2019 [28]	UK	Self-harm	RCT	754	11–17	EQ-5D-3L	UK	Self-report	0.68 (0.27)
Le, 2021 [24]	Australia	Self-harm Suicide ideation	Cross-sectional	Self-harm: n = 328 Suicide ideation n = 220	11–17	CHU9D	Australian (adolescents)	Self-report	Self-harm: 0.57 (0.26); Suicide ideation 0.54 (0.26)
12. General mental health problems									
Le, 2019 [54]	Australia	Mental health problems screened by Kessler-10	RCT	413	18–25	AQoL4D	Australian	Self-report	Both intervention and control groups: 0.56 (0.26)
Furber, 2015 [55]	Australia	Having an open episode of mental health care within the last 6 weeks Mental health problems	Cross-sectional	200	5–17 (11.71)	CHU9D	UK (adults) and Australian (Adolescents) tariffs	Proxy report (caregivers)	UK tariff: 0.803 (0.117); Australian tariff: 0.739 (0.145)
Le 2021 [24]	Australia	Mental health diagnosis by Diagnostic Interview Schedule for Children (DISC-IV)	Cross-sectional	415	11–17	CHU9D	Australia (adolescents)	Self-report	0.70 (0.24)
Wolf, 2021 [59]	Denmark	Mental health problems screened by Strengths and Difficulties Questionnaire	RCT	396	6–15 (10.7)	CHU9D	UK (adults) and Australian (Adolescents) tariffs	Proxy report (parents)	UK tariff: 0.804 (0.121); Australian tariff: 0.629 (0.214)

#### Study population and conditions

Table 2 presents an overview of the 12 types of MHPs explored. Overall, 35 out of 38 studies assessed only one MHPs while three studies assessed more than one MHP such as 8 MHPs [24], or 3 MHPs [25, 26]. The most studied MHP was ADHD (n = 10), followed by depression (n = 9), anxiety disorders (n = 4), substance use (n = 4), undefined psychiatric disorders (n = 4), psychosis (n = 3), personality disorders (n = 2), behaviour disorders (n = 2), PTSD (n = 2), self-harm (n = 2), internalizing problems (n = 1), and general MHPs (n = 3). The sample size in included studies varied from 43 [27] to 754 [28]. The included studies were from the UK (n = 14) [28–39], the Netherlands (n = 6) [40–45], Finland (n = 3) [46–48], Sweden (n = 2) [26, 49, 50], Germany (n = 1) [51, 52], Australia (n = 4) [24, 53–55], US (n = 3) [27, 56, 57], and one study each from Brazil [58], Denmark [59], Norway [60], one study in Ireland and UK [25], and one in UK and US [61].

**Table 2:** Elicitation techniques used by MHPs

	EQ-5D-3L	EQ-5D-5L	EQ-5D-Y	CHU9D	HUI2	HUI3	QWB	SF-6D	16D	AQoL4D	SG	TTO
ADHD	3 [30, 40, 61]		2 [29, 31]	3 [24, 29, 31]	2 [25, 58]	2 [25, 58]					2 [27, 30]	1 [66]
AD	1 [41]		[26]	3 [24, 32, 53]								
Depression	5 [34, 35, 37, 56, 57]	1 [33]	1 [26, 36]	1 [24]	3 [36, 56, 57]	2 [56, 57]	2 [56, 57]	2 [56, 57]				
IPs						1 [50]						
PD	2 [38, 63]											
BD	1 [43]			1 [24]	1 [25]	1 [25]						
PTSD		2 [52, 64]							1 [60]			
Substance use	2 [44, 45]	1 [65]		1 [24]								
Psychiatric disorders	1 [39]		1 [26]		1 [25]	1 [25]						
Psychosis									3 [46–48]			
Self-harm	1 [28]			1 [24]								
MHPs				3 [24, 55, 59]						[54]		

*AD* anxiety disorder, *AN* anorexia nervosa, *IPs* internalizing problems, *PTSD* posttraumatic stress disorder, *ADHD* attention-deficit/hyperactivity disorder, *PD* personality disorder, *BD* behaviour problems, *MHPs* general mental health problems, *SG* standard gamble, *TTO* time trade-off

#### Age range

**Fig. 2:**
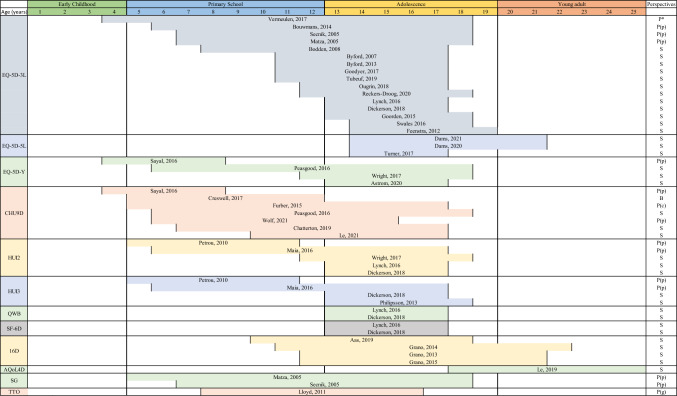
Elicitation methods by age range and perspectives. Notes: P* = Proxy report (parents, professionals and children); P(p) = Proxy-report (parents); P(c) = Proxy report (caregivers); P(g) = Proxy-report (general public); S = Self-report; B = Both self-report and Proxy-report

Figure 2 presents the age range of children and young population in included studies. The age range explored was between 4 and 25 years. EQ-5D-3L was used for a wider age range between 4–19 years. EQ-5D-5L was used mainly for youth aged 14–21. EQ-5D-Y was used for participants aged 4–18. CHU9D was used for children aged between 4 and 17 years. HUI2 and HUI3 were used for participants aged between 5 and 18 years. QWB and SF-6D were used only for adolescents aged 13–18 years. In terms of studies employing direct elicitation techniques, the SG was used for participants aged 5–18 years while TTO for those aged 9–16 years. For samples including mixed adolescents and adults, we found EQ-5D-3L was used for samples aged 14–19 years [63], EQ-5D-5L in samples aged 14–21 years [52, 64] and 15–19 years [65], 16D in samples aged between 11–22 years [46–48].

#### Valuation methods

Table 2 summarises valuation methods and instruments used in the included studies. Most studies (n = 36) adopted the indirect valuation method, while two used the direct valuation method including SG [27] and TTO [66] which were used only for ADHD. Among 10 MAUIs used as the indirect valuation method, EQ-5D-3L was the most frequently used (n = 16) in 9 types of MHPs, followed by CHU9D (n = 13), HUI2 (n = 7), HUI3 (n = 6), EQ-5D-Y (n = 5), EQ-5D-5L (n = 3), 16D (n = 4), QWB (n = 2), SF-6D (n = 2), and AQoL4D (n = 1). Several studies used more than one MAUI including two studies that used both EQ-5D-Y and CHU9D [29, 31], two using HUI2 and HUI3 [25, 58], one using EQ-5D-Y and HUI2 [36], and two studies using four MAUIs (HUI2, HUI3, EQ-5D-3L, QWB, SF6D) [56, 57]. One study used both direct (SG) and indirect valuation methods (EQ-5D-3L) [30].

#### Algorithms

EQ-5D-3L was used in five studies with the Dutch (adult) algorithm [40, 44, 45, 63], two with the German (adult) algorithm [52, 64], and four with the UK adult algorithm [30, 35, 38, 41]. EQ-5D-5L was used with the UK adult algorithm [33]. CHU9D was used with the UK adult algorithm in four studies [29, 31, 32, 59], and the Australian adolescent algorithm [53, 59]. HUI2 and HUI3 were used with the Canadian adult algorithm [56–58] while HUI2 was used with the UK adult algorithm in one study [25]. SF-6D was used with the UK adult [56, 57]. Only one study [31] reported HSUVs for EQ-5D-Y using the UK adult algorithm while another study using EQ-5D-Y did not report HSUVs [26] possibly due to the lack of a corresponding value set at the time [23].

#### Perspectives

Figure 2 summarises the use of elicitation methods and MAUIs used by perspectives. Twenty-eight studies administered the MAUI to children and youth using self-report while 16 studies used proxy-report (either a direct elicitation method and/or MAUIs) including parents (n = 13), caregivers (n = 1), teachers (n = 1), professionals (n = 1), general public (n = 1), and children and youth (i.e. evaluating bespoke health states description) (n = 1). Proxy-report was mainly adopted in studies exploring ADHD (8 out 10 studies) while all studies exploring depression, post-traumatic stress disorders, cannabis use disorder, psychosis and self-harm adopted self-report. Studies using the direct valuation methods such as SG [27, 30] and TTO [66] all adopted proxy-report. Indirect valuation methods were used with proxy-report for children as young as 4 years old [31] and self-report in children as young as 5 years old [32].

### Reported utility values by MHPs

**Fig. 3:**
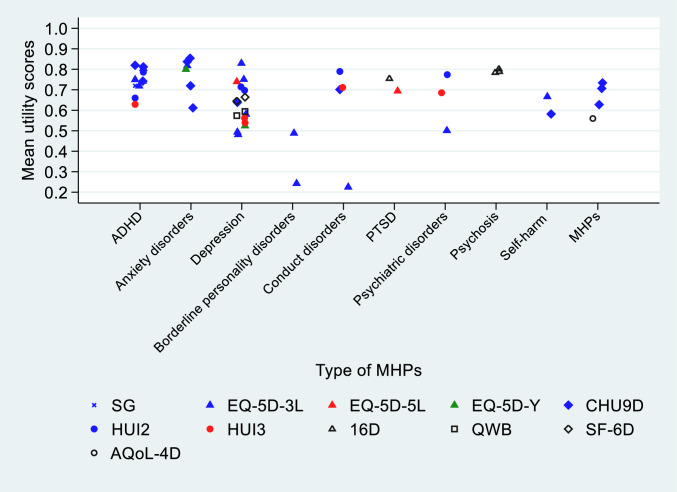
Reported HSUVs by type of elicitation tools across MHPs. Notes: MHPs having HSUVs derived from only one study were excluded (internalising problems, medicine use, delinquency, tobacco use, other drug used disorders, avoidant personality disorder, personality disorder, depressive personality disorder, and obsessive-compulsive personality disorder)

Figure 3 shows the HSUVs derived by different elicitation methods and MAUIs used across MHPs that were explored in more than one study. Across all generated HSUVs by MHPs, Disruptive Behaviour Disorder had the lowest HSUV of 0.06 [43] while cannabis use disorder was associated with the highest HSUVs of 0.88 [44].

HSUVs reported for ADHD ranged from 0.444 [66] to 0.897 [31]. Only two studies reported HSUVs associated with severity levels, in which HSUVs for mild ADHD were 0.71 and 0.787, for severe ADHD 0.48 and 0.444 [27, 66]. HSUVs reported for anxiety disorders ranged from 0.62 [53] to 0.88 [32], and for depression from 0.495 [34] to 0.81 [57]. HSUVs for post-traumatic stress disorder range between 0.70 [52, 64] to 0.755 [60], for borderline personality disorder from 0.236 [38] to 0.49 [63]. HSUVs associated with undefined psychiatric disorders ranged from 0.5 [39] to 0.782 [25], for psychosis from 0.7992 [47] to 0.80 [46], for self-harming from 0.57 to 0.68 [28], for alcohol use from 0.82 [45] to 0.73 [24], for conduct disorders from 0.11 [43] to 0.802 [25], and for general MHPs from 0.56 [54] to 0.804 [59].

HSUVs for the following MHPs were derived from only one study each where HSUVs associated with internalising problems were 0.71 [50], with medicine use and delinquency in adolescents with MHPs being 0.81 and 0.82, respectively [45]. Furthermore, HSUVs of 0.49 were reported for avoidant personality disorder, 0.70 for personality disorder not otherwise specified, 0.34 for depressive personality disorder, 0.50 for obsessive–compulsive personality disorder [63], 0.52 for oppositional defiant disorder [43], 0.54 for suicide ideation [24].

Across MAUIs, Fig. 3 shows that the generated HSUVs vary significantly among the most frequently explored types of MHPs while the variations could not be overserved obviously in MHPs explored by fewer studies (n < 3). Specifically, CHU9D yielded generally higher mean HSUV scores while HUI2 and HUI3 yielded lower scores than other MAUIs in ADHD. Interestingly, EQ-5D-3L generated the widest range of HSUVs from the lowest HSUV of 0.495 [34, 57] to the highest of 0.81 among all included scores in depression. Similarly, CHU9D generated both the lowest HSUV of 0.62 [53] to the highest HSUV of 0.88 [32] in anxiety disorders.

A wide variation in reported HSUVs between self- and proxy-report was also observed (Supplementary Document 3). While self-reported HSUVs for anxiety disorders vary significantly, most of them (ranging from 0.62 [53] to 0.87 [32]) are general lower than proxy-reported HSUVs (0.85 [32]). In contrast, proxy-reported HSUVs for undefined psychiatric disorders are generally higher than self-reported ones. In conduct disorders, proxy-reported HSUVs were generally higher than self-reported ones except for one proxy-reported HSUV being the lowest score [43]. Among all included MHPs, depression has the widest range of HSUVs which were all derived from self-report.

A meta-analysis was considered inappropriate given the heterogeneity of reported HSUVs caused by different elicitation methods and MAUIs used, scoring algorithms, age range, country of research, and study designs.

### Psychometric performance

Only nine studies assessed the psychometric performance of MAUIs used in youth mental health population in which five studies assessed EQ-5D-3L [35, 40, 56, 57, 61], two studies assessed EQ-5D-5L [26, 64], two studies assessed CHU9D [55, 59], and two studies [56, 57] assessed four MAUIs (EQ-5D-3L, QWB, SF-6D, HUI2 and HUI3). Table 3 reports the positive evidence, mixed evidence and no evidence for each psychometric property per MAUI, followed by the clinical population assessed.

**Table 3:** Psychometric performance

Study reference	Type of disorder tested	N	Age	Algorithm	Perspectives	Discriminant validity	Convergent validity	Responsiveness	Feasibility	Test–retest reliability	Internal consistency
EQ-5D-3L											
Matza, 2005 [61]	ADHD	126	7–18	UK (adults)	Proxy-report (parent)	Y	Y				
Bouwmans, 2014 [40]	ADHD		6–18	Dutch (adults)	Proxy (parent)	Y		Y			
Byford, 2013 [35]	Depression	199	11–17	UK (adults)	Self-report	Y	+ -	Y			
Lynch, 2016	Depression	392	13–17	NA	Self-report	Y					
Dickerson, 2018 [57]	Depression	376	13–17	NA	Self-report		Y	Y			
EQ-5D-5L											
Dams, 2021 [64]	PTSD	87	14–21	German (adults)	Self-report	Y	Y	N		Y	
EQ-5D-Y-5L											
Astrom, 2020 [26]	Psychiatric disorders	52	13–17	Dimension	Self-report	Y	+ -		Y		
CHU9D											
Furber, 2015 [55]	MHPs	200	5–17	UK (adults) and Australian (Adolescents) tariffs	Proxy report (caregivers)		Y				Y
Wolf, 2021 [59]	MHPs	396	6–15	UK (adults) and Australian (Adolescents) tariffs	Proxy (parent)	Y	Y	Y			
QWB											
Lynch, 2016	Depression	392	13–17	Canada	Self-report	Y					
Dickerson, 2018 [57]	Depression	376	13–17	Canada	Self-report		Y	Y			
SF-6D											
Lynch, 2016	Depression	392	13–17	UK (adults)	Self-report	+−					
Dickerson, 2018 [57]	Depression	376	13–17	UK (adults)	Self-report		Y	Y			
HUI3											
Lynch, 2016	Depression	392	13–17	Canada	Self-report	Y					
Dickerson, 2018 [57]	Depression	376	13–17	Canada	Self-report		Y	Y			
HUI2											
Lynch, 2016	Depression	392	13–17	Canada	Self-report	Y					
Dickerson, 2018 [57]	Depression	376	13–17	Canada	Self-report		Y	Y			

Y: psychometric performance was significant, N: Psychometric performance was not significant, +−: Psychometric performance was inclusive

*MHPs* General mental health problems

No study assessed all psychometric properties investigated in this review. Overall, seven studies assessed discriminant validity, six studies assessed convergent validity, five studies assessed responsiveness, one study each assessed test–retest validity and feasibility. Three studies assessed the psychometric performance of MAUIs using proxy-report while six studies used self-report.

For EQ-5D-3L, the review found evidence of discriminating validity, convergent validity and responsiveness from both self-report [35, 56, 57] and proxy (parent) report [40, 61]. Whilst there is evidence of the discriminant validity, convergent validity, test–retest validity and feasibility in using EQ-5D-5L for youth population with mental health [64], the review did not find evidence of responsiveness for EQ-5D-5L [64]. For EQ-5D-Y-5L, one study using self-report showed evidence to support its feasibility, discriminant validity, and test–retest validity, however, the evidence of its convergent validity is mixed [26]. Regarding CHU9D, two included studies using proxy-report (parent) found evidence of the discriminant validity, convergent validity, internal consistency, and responsiveness of the dimensions of CHU9D [55, 59]. One study also provided evidence of the discriminant validity, convergent validity, and responsiveness of CHU9D using both UK (adults) and Australian (adolescents) algorithms [59]. For QWB, SF-6D, HUI2&3, the review found that these MAUIs have discriminant validity, convergent validity, and responsiveness [56, 57].

## Discussion

This paper, for the first time, provided a systematic review of evidence on (1) the HSUVs of various MHPs, (2) the current practice to generate HSUVs, and (3) the psychometric performance of MAUIs used in children and youths with MHPs. The review found 38 studies reporting HSUVs for 12 types of MHPs in children and youths aged between 4 and 25 years across 12 countries between 2005 and October 2021. Among 12 types of MHPs reported in the review, depression and ADHD are the most explored MHPs, with 10 studies each. Overall, the review found that Disruptive Behaviour disorder has the lowest HSUV of 0.06 from children in primary education valuing vignettes in a cross-sectional study [43] while cannabis use disorder was associated with the highest HSUVs of 0.88 from an intervention study [44]. Moreover, the indirect valuation method using MAUIs (used in 95% of included studies) is the most frequently used approach to generate HSUVs for all 12 MHPs while only two studies using the direct valuation method was focused on ADHD. Of the ten MAUIs found in this review, the most frequently used was EQ-5D-3L, used in 15 out of 38 studies across eight out of 12 types of MHPs. CHU9D, the only MAUIs developed for use in childhood specific populations was used in 13 out of 38 studies across seven out of 12 MHPs. However, the review found no measure that has sufficient evidence of psychometric performance in children and youths with MHPs.

Heterogeneity was noticeable across the included studies. The variations, mainly caused by different elicitation methods and MAUIs used, scoring algorithms, age range, country of research, and study designs, have made it difficult to derive an estimate of the overall effect. For example, using samples of 11–17 years old adolescents at risk of self-harm, one study reported an HSUV of 0.68 using EQ-5D-3L with the UK adult tariff [28] while another study reported an HSUV of 0.57 using CHU9D with the Australian adolescent tariff [24]. Even using the same clinical sample, different MAUIs produced different HSUVs, for example, an HSUV of 0.56 derived from HUI3 while an HSUVs of 0.81 from EQ-5D-3L based on the same sample of adolescents with depression [57]. These findings are consistent with results from adult literature where different MAUIs produced different scores even in the same person [67]. Furthermore, only one study reports the populations norms for several MHPs [24], none of studies compared HSUVs with their corresponding populations norms. Thus, future research may benefit from such comparison.

We found some MAUIs were used for samples with the age range different from their recommended age range. Specifically, EQ-5D-3L, an adult-specific MAUI, was used in children aged as young as 4 years through proxy-report [31] or as young as 8 years old using self-report [41]. While the recommended ages for 16D are 12–15 years [10], the review found that the 16D was used in adolescents as young as 10 years old [60] or as old as 22 years [48]. We noted that studies may use measures for sample outside of the recommended age range as a way to save time and resources, or to facilitate comparisons across different age groups. However, transitioning between child and adolescent/youth populations can be challenging as there are significant developmental differences between these age groups [68]. Therefore, we urge future research to consider the potential limitations and accuracies that may result from this approach.

This review found limited evidence of the psychometric performance of MAUIs used in children and youths with MHPs with only nine studies covering eight MAUIs in five types of MHPs. Of these MAUIs, more studies assessed the psychometric performance of EQ-5D-3L than other measures. Only CHU9D was assessed using a sample with general MHPs while other MAUIs were assessed using a specific MHP samples. Specifically, EQ-5D-3L was assessed in ADHD and depression, EQ-5D-5L was assessed in PTSD, EQ-5D-Y-5L was assessed in psychiatric disorders, and QWB, SF-6D, HUI2&HUI3 were assessed in depression. Furthermore, we found that MAUIs were used in MHPs where evidence on their psychometric performance is lacking, such as the use of EQ-5D-3L in personality disorder [38, 63], behaviour disorders [43], substance used [44, 45, 65] and psychiatric disorders [39].

Evidence on the psychometric performance of MAUIs used in children and youths with MHPs is not conclusive, which makes it difficult to recommend one MAUI over another. Although the best psychometric evidence exists for EQ-5D-3L and CHU9D in terms of discriminant validity, convergent validity and responsiveness, other psychometric properties remain unclear. For example, both CHU9D and EQ-5D-3L have not been assessed regarding their feasibility, test–retest reliability, and interrater reliability. The evidence of other MAUIs was found either mixed or limited in number of studies and type of MHPs explored. Specifically, EQ-5D-5L was found to lack responsiveness while EQ-5D-Y-5L showed inconsistencies in its ability to converge with other measures. Although the psychometric performance of EQ-5D-5L and EQ-5D-Y-5L may look alarming, it is worth noting that the evidence was derived from one study per measure with small samples less than 100 which may limit the statistical power of the results. The findings on the convergent and discriminant validity of EQ-5D-3L, SF-6D and HUI3 in adolescents with depression align with findings in the adult depression literature [14, 15]. The limited evidence in terms of type of MHPs explored, extensive psychometric properties, number of studies with sufficient samples urges further research to provide comprehensive picture of the psychometric properties of all measures used in children and youths with MHPs.

Indeed, various aspects of MAUI psychometric assessments need further attention. Specifically, we found limited or no evidence in test–retest reliability, internal consistency, and interrater reliability of MAUIs. Various validity tests such as face validity and content validity would also contribute to more conclusive evidence of the psychometric performance of MAUIs in children and youths with MHPs. Lastly, although MAUIs were used across different countries using different languages, the review noted that the cultural validity of MAUIs has not been investigated.

Limitations of our study include the inability to conduct a meta-analysis of reported HSUVs due to the heterogeneity of the included studies and the small number of studies per MHP, which would otherwise provide an overview picture of the impact of MHPs on HSUVs and the associated disease burden [22]. Non-English studies and grey literature were excluded, which may limit the comprehensiveness of our review. Furthermore, following previous systematic reviews [10, 11, 19, 23], we did not conduct quality assessment of the included studies as well as the appropriateness of the statistical analyses undertaken in included studies, however results of included studies were discussed in consideration of sample sizes.

## Conclusion

This review provides an overview of HSUVs of various MHPs, the current practice to generate HSUV, and the psychometric performance of MAUIs used in children and youths with MHPs. We report limited evidence on the psychometric performance of MAUIs in terms of psychometric properties assessed, type of MHPs explored, number of studies per measure and sufficient sample sizes. This highlights the need for more rigorous and extensive psychometric assessments to produce conclusive evidence on the suitability of MAUIs used in this area.

### Supplementary Information

Below is the link to the electronic supplementary material.

Supplementary file1 (DOCX 46 KB)

## Data Availability

Data sharing is not applicable to this article as no new data were created or analyzed in this study.
