# Supplementary material for: A systematic review of health state utility values and psychometric performance of generic preference-based instruments for children and adolescents with mental health problems
Source: Qual Life Res. 2023 May 26;32(11):3005–26. doi: 10.1007/s11136-023-03441-x (PMC10522744; doi:10.1007/s11136-023-03441-x)
Supplement: Supplementary file 1 — Supplementary file1 (DOCX 46 KB) [file 11136_2023_3441_MOESM1_ESM.docx]

**A systematic review of Health State Utility Values for Children and adolescents with mental health problems**

# Search Terms

|  |  | Ovid MEDLINE(R) and In-Process, In-Data-Review & Other Non-Indexed Citations <1946 to October 25, 2021> |
| --- | --- | --- |
| Indirect preference elicitation methods | 1 | (Multiattribute or multi-attribute).mp. |
|  | 2 | (EQ 5D$ or EQ5D$).mp. |
|  | 3 | (EuroQol$ or Euro Qol$).mp. |
|  | 4 | SF-6D.mp. |
|  | 5 | (SF 12$ or SF12$ or Short-form 12$ or Short-form12$ or MOS 12 or MOS12).mp. |
|  | 6 | (SF 36$ or SF36$ or Short-form 36$ or Short-form36$ or MOS 36 or MOS36).mp. |
|  | 7 | (RAND36$ or RAND 36$).mp. |
|  | 8 | (VR36$ or VR 36$).mp. |
|  | 9 | (Health Utilities Index or Health Utility Index).mp. |
|  | 10 | (HUI-2 or HUI2 or HUI-3 or HUI3).mp. |
|  | 11 | quality of wellbeing scale?.mp. |
|  | 12 | QWB$.mp. |
|  | 13 | assessment of quality of life.mp. |
|  | 14 | AQoL$.mp. |
|  | 15 | 17d.tw. |
|  | 16 | 16d.tw. |
|  | 17 | 15d.tw. |
|  | 18 | (CHU 9D$ or CHU9D$ or CHU-9D).mp. |
|  | 19 | mapping.mp. |
|  | 20 | valuation model.tw. |
|  | 21 | valuation model?.tw. |
|  | 22 | 1 or 2 or 3 or 4 or 5 or 6 or 7 or 8 or 9 or 10 or 11 or 12 or 13 or 14 or 15 or 16 or 17 or 18 or 19 or 20 or 21 |
| Direct preference elicitation methods | 23 | Trade off?.mp. |
|  | 24 | standard gamble?.mp. |
|  | 25 | analogue scale?.mp. |
|  | 26 | visual analogue scale.mp. |
|  | 27 | VAS scale.mp. |
|  | 28 | discrete choice experiment?.mp. |
|  | 29 | DCE?.mp. |
|  | 30 | person trade off.mp. |
|  | 31 | scaling method?.mp. |
|  | 32 | magnitude estimation.mp. |
|  | 33 | contingency valuation?.mp. |
|  | 34 | CV?.mp. |
|  | 35 | best worst scaling?.mp. |
|  | 36 | bws?.mp. |
|  | 37 | (elicit$ adj8 (preference? or valu$ or view?)).mp. |
|  | 38 | (valuation? adj3 stud$).mp. |
|  | 39 | direct valuation?.mp. |
|  | 40 | 23 or 24 or 25 or 26 or 27 or 28 or 29 or 30 or 31 or 32 or 33 or 34 or 35 or 36 or 37 or 38 or 39 |
| Youth | 41 | (girl or girls).tw,kw. |
|  | 42 | (boy or boys).tw,kw. |
|  | 43 | adolescen*.tw,kw. |
|  | 44 | (school* or teen* or youth* or peer* or student*).tw,kw. |
|  | 45 | (child* or kid or kids or pediatric* or paediatric*).tw,kw. |
|  | 46 | (young person or young people or young adult*).tw,kw. |
|  | 47 | 41 or 42 or 43 or 44 or 45 or 46 |
| Utility values | 48 | Quality-Adjusted Life Years/ |
|  | 49 | Value of Life/ |
|  | 50 | wellbeing.mp. |
|  | 51 | qwb.mp. |
|  | 52 | (quality and wellbeing).mp. |
|  | 53 | (utility or utilities or disutility or disutilities).mp. |
|  | 54 | quality of life.mp. |
|  | 55 | ((preference-based or generic) adj8 (instrument? or measure?)).mp. |
|  | 56 | (health status adj3 (measure? or utility$)).mp. |
|  | 57 | health status indicator.mp. |
|  | 58 | (health state adj8 preference?).mp. |
|  | 59 | (health adj5 preference?).mp. |
|  | 60 | (utilit$ adj10 health state?).mp. |
|  | 61 | (utilit$ adj10 health status).mp. |
|  | 62 | (elicit$ adj5 utilit$).mp. |
|  | 63 | ((valuation? or value?) adj5 state?).mp. |
|  | 64 | ((valuation? or value?) adj8 health state?).mp. |
|  | 65 | ((preference? or valu$ or utility$) adj8 state?).mp. |
|  | 66 | preference scale?.mp. |
|  | 67 | (preference? adj3 (public or health or social or societal or society)).mp. |
|  | 68 | (health adj3 reference?).mp. |
|  | 69 | (valu$ adj2 (societal or social)).mp. |
|  | 70 | social values.mp. |
|  | 71 | preference? elicitation?.mp. |
|  | 72 | (preference adj5 valu$).mp. |
|  | 73 | tariff?.tw. |
|  | 74 | value? set?.tw. |
|  | 75 | valuation set?.tw. |
|  | 76 | preference weight?.tw. |
|  | 77 | value of life/ec [economics] |
|  | 78 | 48 or 49 or 50 or 51 or 52 or 53 or 54 or 55 or 56 or 57 or 58 or 59 or 60 or 61 or 62 or 63 or 64 or 65 or 66 or 67 or 68 or 69 or 70 or 71 or 72 or 73 or 74 or 75 or 76 or 77 |
| Mental health | 79 | exp Mental Health/ |
|  | 80 | exp Mental Disorder/ |
|  | 81 | exp Depression/ |
|  | 82 | ((anxi* adj3 disorder$) or (anxi* adj3 symptom$) or generalized anxiety disorder or GAD or social anxiety).ti,ab,kf. |
|  | 83 | (stress or distress or PTSD or PTSS or (posttraumatic adj1 symptom$) or emotion* trauma or trauma-related disorder$ or traumatic neurosis or mental distress or emotion* distress or motion* stability or (psycho* adj1 distress*) or (psychiat* adj1 distress*)).ti,ab,kf. |
|  | 84 | (panic* or phobi*).ti,ab,kf. |
|  | 85 | (depressive disorder or (depressi* adj3 disorder$) or (depressi* adj3 symptom$) or (depressi* adj3 episode$)).ti,ab,kf. |
|  | 86 | ((mental adj1 health) or (mental adj1 disorder$) or (mental adj1 illness) or (mental adj1 problem$) or (psycho* adj1 problem$) or (psycho* adj1 disorder$) or (psycho* adj1 illness$) or (psychiat* adj1 disorder$) or (psychiat* adj1 illness$)).ti,ab,kf. |
|  | 87 | (Attention-deficit hyperactivity disorder or adhd).ti,ab,kf. |
|  | 88 | Suicid*.ti,ab,kf. |
|  | 89 | behavio?r disorder*.ti,ab,kf. |
|  | 90 | (eating disorders or anorexia or bulimia or disordered eating or binge eating disorder).ti,ab,kf. |
|  | 91 | (schizophrenia or psychosis or psychoses or psychotic disorder or schizophrenic disorder).ti,ab,kf. |
|  | 92 | (bipolar adj5 (disorder or disease or illness)).ti,ab,kf. |
|  | 93 | emotional disorder?.ti,ab,kf. |
|  | 94 | Personality Disorder?.ti,ab,kf. |
|  | 95 | (self-harm or self-injury or self harm or self injury).ti,ab,kf. |
|  | 96 | 79 or 80 or 81 or 82 or 83 or 84 or 85 or 86 or 87 or 88 or 89 or 90 or 91 or 92 or 93 or 94 or 95 |
|  | 97 | 22 and 47 and 78 and 96 |
|  | 98 | 22 and 47 and 78 and 96 |
|  | 99 | limit 98 to english language |
|  | 100 | 40 and 47 and 78 and 96 |
|  | 101 | 40 and 47 and 78 and 96 |
|  | 102 | limit 101 to english language |

# Detailed characteristics of included studies

| **Characteristics** | **Number of studies** | **(%)** |
| --- | --- | --- |
| **Country of Origin** |  |  |
| Australia | 4 | 10.5 |
| Brazil | 1 | 2.6 |
| Denmark | 1 | 2.6 |
| Finland | 3 | 7.9 |
| Germany | 1 | 2.6 |
| Netherlands | 6 | 15.8 |
| Norway | 1 | 2.6 |
| Spain | 0 | 0.0 |
| Sweden | 2 | 5.3 |
| UK | 14 | 36.8 |
| US | 3 | 7.9 |
| US & UK | 1 | 2.6 |
| UK & Ireland | 1 | 2.6 |
| **Years conducted** |  |  |
| 2021 | 3 | 7.9 |
| 2020 | 3 | 7.9 |
| 2019 | 4 | 10.5 |
| 2018 | 2 | 5.3 |
| 2017 | 5 | 13.2 |
| 2016 | 5 | 13.2 |
| 2015 | 3 | 7.9 |
| 2014 | 2 | 5.3 |
| 2013 | 3 | 7.9 |
| 2012 | 1 | 2.6 |
| 2011 | 1 | 2.6 |
| 2010 | 1 | 2.6 |
| 2009 | 0 | 0.0 |
| 2008 | 1 | 2.6 |
| 2007 | 1 | 2.6 |
| 2006 | 0 | 0.0 |
| 2005 | 3 | 7.9 |
| **Type of study** |  |  |
| Cross-sectional | 20 | 52.6 |
| RCT | 18 | 47.4 |
| **Perspectives** |  |  |
| Self-report | 28 | 73.7 |
| Proxy-report (parents) | 13 | 34.2 |
| Proxy-report (professionals) | 1 | 2.6 |
| Proxy-report (Children & Adolescents) | 1 | 2.6 |
| Proxy report (general public) | 1 | 2.6 |
| Notes: Percentages do not add up to 100% in case of "perspective" as some studies reported more than just one type of perspectives. | | |

1. Reported HSUVs by perspectives across MHPs
2.

Reported HSUVs by perspectives across MHPs

Notes: MHPs having HSUVs derived from only one study were excluded (internalising problems, medicine use, delinquency, tobacco use, other drug used disorders, avoidant personality disorder, personality disorder, depressive personality disorder, and obsessive-compulsive personality disorder)
